# Caregivers of Older Adults With Cognitive Impairment

**Published:** 2009-03-15

**Authors:** Erin DeFries, Lisa C. McGuire, Elena M. Andresen, Babette A. Brumback, Lynda A. Anderson

**Affiliations:** Department of Epidemiology and Biostatistics, College of Public Health and Health Professions, University of Florida; Centers for Disease Control and Prevention, Atlanta, Georgia; University of Florida, Gainesville, Florida; University of Florida, Gainesville, Florida; Centers for Disease Control and Prevention, Emory University, Atlanta, Georgia

## Abstract

**Introduction:**

Because of the growing number of caregivers and the awareness of related health and quality-of-life issues, caregiving has emerged as an important public health issue. We examined the characteristics and caregiving experiences of caregivers of people with and without cognitive impairment.

**Methods:**

Participants (n = 668) were adults who responded to the 2005 North Carolina Behavioral Risk Factor Surveillance System. Caregivers were people who provided regular care to a family member or friend aged 60 years or older either with or without cognitive impairment (ie, memory loss, confusion, or Alzheimer's disease).

**Results:**

Demographic characteristics of caregivers of people with cognitive impairment were similar to those of caregivers of people without cognitive impairment. However, compared with caregivers of people without cognitive impairment, caregivers of people with cognitive impairment reported higher levels of disability, were more likely to be paid, and provided care for a longer duration. Care recipients with cognitive impairment were more likely than care recipients without cognitive impairment to be older, have dementia or confusion, and need assistance with memory and learning.

**Conclusion:**

State-level caregiving surveillance is vital in assessing and responding to the needs of the growing number of caregivers.

## Introduction

The expansion of the aging population in the United States is well documented. According to census estimates, 1 in every 5 (20.7%) people in the United States will be aged 65 or older by 2050, compared with 1 in 10 (10.4%) in 2000 (1). Because disability increases with age (2), the number of people who need assistance with activities of daily living (ADL) (eg, bathing) and instrumental activities of daily living (IADL) (eg, meal preparation) will continue to increase as the population ages. Historically, family members and friends have provided most of the assistance needed for the aging population in the United States. Approximately two-thirds of community-dwelling adults who need assistance with ADL rely on family members and friends alone to meet their needs (3).

Informal caregiving is a component of health, social, and aging services infrastructures (4-7). Although no universally accepted definition of informal caregiving exists, it is commonly understood as providing assistance to a family member or friend in a nonprofessional, usually unpaid, role to support the capacity of an individual to remain at home in the community for as long as possible (8). An estimated 16% to 30% of Americans provide informal care (9-11). Furthermore, among caregivers of people aged 60 years or older, between 25% and 29% provide assistance to someone with cognitive impairment, a memory problem, or a disorder, such as Alzheimer's disease (10,12).

Aspects of cognition, such as memory, thought, and language, influence a person's ability to interact socially and to function independently (13,14). Cognitive impairment can affect a person's memory as well as the ability to perform daily tasks (15). Caregivers of people with cognitive impairment face challenges common to those of other caregivers, but they also encounter issues unique to the characteristics of the recipient's impairment. Studies have shown that providing care for a person with cognitive impairment is more demanding than caring for someone with physical problems alone, as indicated by reports of higher levels of burden, stress, and depression among caregivers of people with cognitive impairment (4,10,16-19).

Studies of caregivers of people with cognitive impairment have shaped our understanding of specific experiences and outcomes related to caregiving. However, such studies typically focus on a specific group of caregivers and care recipients, such as spousal caregivers, primary caregivers, or those seeking care in a clinic (17,18), which do not represent all caregivers in the population. A consistent source of state-level information on caregiving is needed to adequately assess the population and to plan appropriately for programs and services targeting caregivers. Typically, these services are delivered at the state level. Likewise, surveillance systems such as the Behavioral Risk Factor Surveillance System (BRFSS) provide the opportunity to monitor the burden of cognitive impairment, which is critical to understand the effects of these issues on families and communities in the United States (13,20).

*Healthy People 2010* recommends the use of population-based data for tracking and measuring health indicators over time (21). One of the systems commonly used to monitor *Healthy People* goals is the BRFSS, an annual, list-assisted, random-digit–dialed telephone survey of the noninstitutionalized adult population of the United States and its territories. The BRFSS has been used to survey Americans on health behaviors and risk factors since 1984. Detailed methods have been described elsewhere (22,23), and information about questions, response characteristics, and methods can be found at www.cdc.gov/brfss.

We examined the characteristics of caregivers of people with and without cognitive impairment and the differences in their caregiving experiences.

## Methods

From May through August 2005, an 11-item module of caregiving questions was added to the North Carolina BRFSS (24). These questions were created through collaborative efforts with key national stakeholders as part of a larger pilot study that also involved a follow-back survey of consenting caregivers (24). North Carolina was chosen as the pilot site because the large sample planned for 2005 BRFSS allowed a sufficient number of responses (study plan, n = 5,000) within 4 months. This study was approved by the institutional review board of the University of Florida.

### Measures

The demographic factors of age, race/ethnicity, sex, education, and income were used to characterize caregivers. Age was reported as a categorical variable (18-34,18-34, 35-44, 45-54, 55-64, and ≥65 years). Categories for race/ethnicity (non-Hispanic white; non-Hispanic black; other/multi-race, non-Hispanic; and Hispanic), sex, education level (<high school diploma, high school diploma, and >high school diploma), and annual income (<$25,000; $25,000-$34,999; $35,000-$49,999; $50,000-$74,999; and ≥$75,000) also were reported.

Health-related quality of life of the caregiver was measured by responses to the following 3 core questions: 1) "Now thinking about your physical health, which includes physical illness and injury, for how many days during the past 30 days was your physical health not good?"; 2) "Now thinking about your mental health, which includes stress, depression, and problems with emotions, for how many days during the past 30 days was your mental health not good?"; and 3) "Would you say that in general your health is excellent, very good, good, fair, or poor?" The reliability of these questions is reported elsewhere (25). Social and emotional support was assessed through a single question: "How often do you get the social and emotional support you need?" Life satisfaction was measured by a single question: "In general, how satisfied are you with your life?"

Respondents were characterized as having a disability if they answered yes to either of the 2 following core questions: 1) "Are you limited in any way in any activities because of physical, mental, or emotional problems?" or 2) "Do you now have any health problem that requires you to use special equipment, such as a cane, a wheelchair, a special bed, or a special telephone?" Objective 6-1 of *Healthy People 2010* suggests that these items be used nationally to assess disability (21).

Respondents were classified as caregivers if they replied yes to the following question: "People may provide regular care or assistance to someone who has a long-term illness or disability. During the past month, did you provide any such care or assistance to a family member or friend?" This item was modified from a question asked nationally during the 2000 BRFSS that restricted the definition of caregiver to one who provided care to someone aged 60 or older (9). If respondents provided care for more than 1 person, they were instructed to answer all subsequent questions on the basis of the person for whom they provided the most care. Additionally, caregivers who reported that the care recipient was aged 60 years or older were asked, "Did that person have a problem with memory loss or confusion or a disorder like Alzheimer's disease?" Those who said yes were classified as caregivers of people with cognitive impairment. Because the cognitive impairment question was asked only of caregivers of people aged 60 or older, all analyses were restricted to caregivers of people aged 60 or older.

### Caregiving experience

Caregivers were asked a series of questions about their experiences providing care, which included several components: 1) description of the care recipient, 2) type and duration of care provided, and 3) caregiving intensity. Caregivers provided the following information about the person to whom they provided the most care: age (classified as 60-69, 70-79, 80-89, or ≥90 years), sex, relationship to caregiver (spouse/partner, other family member, nonfamily member, or paid caregiver), and major health problem (26 diagnoses possible). Unless otherwise noted, caregivers were limited to 1 answer choice per question.

Type of care provided was assessed through a single question: "Given this condition, with which two of the following areas does he/she most need your help?" (response options: learning, remembering, and confusion; seeing or hearing; taking care of oneself, such as eating, dressing, bathing, or toileting; communicating with others; moving around; getting along with people; or feeling anxious or depressed). Duration of care included the questions: "For how long have your provided care for him/her?" and "In an average week, how many hours do you provide care for him/her because of his/her long-term illness or disability?" Responses to these questions are reported as months of caregiving and average hours of care provided per week.

A variable was created to quantify caregiving intensity. The intensity variable was adapted from a measure of burden in the National Alliance for Caregiving (NAC) and AARP study that measured activities and time spent in caregiving (10) and was constructed as follows: if respondents chose either "taking care of oneself, such as eating, dressing, bathing, or toileting" or "moving around" (items related to ADL) on the type-of-care question, they were assigned 3 points; if caregivers chose both options, they were assigned 4 points. Average hours of care provided per week were divided into 4 categories (0-8, 9-19, 20-39, and ≥40). Each category counted as 1 to 4 points, respectively. Points from the 2 questions were added and then categorized into a 5-level caregiver intensity variable, in which higher scores indicated higher levels of intensity. We found a moderately strong correlation between the newly created intensity measure and the 5-level NAC/AARP scale (*r* = 0.61), using data from a subset of respondents (n = 329) who participated in a follow-up survey and who answered a full list of questions about ADL and IADL.

### Statistical analysis

All analyses were completed by using SPSS version 14.0 with Complex Samples (SPSS Inc, Chicago, Illinois) to account for the sampling design. Because caregiving module data were collected during only a portion of the year (May-August 2005), we adjusted the final weights so that the 4-month period of data collection represented the entire North Carolina population. Statistical analyses using the full 2005 North Carolina BRFSS weights and the reweighting that accounted for the 4-month sample yielded similar results, but we report only the reweighted results. We report means and frequencies as well as 95% confidence intervals. We used independent-sample *t* tests to compare means and χ^2^ tests to compare frequency measures. To test for trends across ordered categorical variables (age, income, education, and intensity), logistic regression models were fit in SPSS wherein the outcome was caregiver status (caring for a person with or without cognitive impairment), and each categorical item was included as the exposure variable, coded in 1-point increments (ie, 1, 2, 3 . . .). The trend test provided a global *P* value for the trend across ordered levels of a variable rather than individual *P* values for each level of the variable. This method generalizes the Cochran-Armitage trend test (26) for use with complex survey data (27). Differences were considered significiant at *P* < .05.

## Results

In total, 5,681 people responded to the caregiver question, of which 895 (15.4% weighted) were caregivers. Of these, 672 reported caring for someone aged 60 or older, and 668 answered the cognitive impairment question; the other 4 respondents were excluded from our analyses because they could not be classified as caregivers of persons with or without cognitive impairment. There were 279 caregivers of people with cognitive impairment (41.5% weighted) and 389 caregivers of people without cognitive impairment (58.5% weighted).

No statistically significant differences were found by age, race/ethnicity, sex, level of education, annual household income, healthy days, self-rated health, social support, or life satisfaction between caregivers of people with and without cognitive impairment (Table 1). A significantly higher proportion of caregivers of people with cognitive impairment had a disability; 24.0% of caregivers of people with cognitive impairment indicated they had a disability compared with 16.1% of caregivers of people without cognitive impairment (*P* = .03). Specifically, 23.4% of caregivers of people with cognitive impairment reported their activities were limited by physical, mental, or emotional problems compared with 15.1% of caregivers of people without cognitive impairment (*P* = .02).

Caregivers of people with cognitive impairment differed significantly from other caregivers in care-recipient attributes and the type of care provided (Table 2). Care recipients with cognitive impairment were significantly older than care recipients without cognitive impairment (*P* = .001), but they were no more likely to be women. Caregivers of people with cognitive impairment were significantly more likely to report being paid than were caregivers of people without cognitive impairment (*P* < .001), although the percentage was low for both groups. Caregivers of people with cognitive impairment were significantly more likely to report that the person they care for had dementia than were caregivers of people without cognitive impairment (*P* < .001), although caregivers of people without cognitive impairment were significantly more likely to report that the person they care for had cancer (*P* = .002) or heart disease (*P* = .03) than were caregivers of people with cognitive impairment. Caregivers of people with cognitive impairment were significantly more likely to report that the people they care for need help with "learning, remembering, confusion" and significantly less likely to report that the people they care for need help with "moving around" than caregivers of people without cognitive impairment (*P* < .001 for both). Caregivers of people with cognitive impairment provided care for a significantly longer period of time than did caregivers of people without cognitive impairment (*P* = .001). No significant differences were found between the 2 caregiver groups for hours of care provided per week or for caregiving intensity.

## Discussion

We found that more than 41% of self-identified caregivers of people aged 60 years or older reported a cognitive impairment in the person for whom they provided care. This percentage is considerably higher than those reported in previous caregiver surveys, such as the NAC/AARP survey that reported a rate of 25% (10). Both the North Carolina BRFSS caregiver module and the NAC/AARP survey were conducted during a 4-month interval; queried respondents using a closed-end question to determine whether the person they cared for had Alzheimer's disease, dementia, or other mental confusion; and relied on the caregiver's assessment rather than a medical diagnosis. However, these surveys varied in terms of respondent eligibility and the age of the care recipient. The 25% prevalence of cognitive impairment (ie, Alzheimer's, dementia, or mental confusion) from the NAC/AARP survey was based on care recipients aged 50 or older; we collected data on care recipients aged 60 years or older. Given that the risk of cognitive impairment and dementia increases with age (14), the prevalence of caregiving for people with such impairments may be higher among older populations of care recipients. The NAC/AARP study included only caregivers who assisted with at least 1 ADL or IADL, yielding a sample of caregivers who potentially provided care to more people who had disabilities than did caregivers in our study. Our study was limited to a single state, whereas the NAC/AARP was a national survey, and the prevalence of cognitive impairment may vary in the United States. For example, the Reasons for Geographic and Racial Differences in Stroke Study showed regional variations in the incidence of stroke and identified a "stroke belt" located in several states in the southeastern United States (28). Similar regional variation in cognitive impairment may exist.

Caregivers of people with cognitive impairment were more likely than caregivers of people without cognitive impairment to have a disability and to report that their activities were limited by their disability. Furthermore, many of the caregivers themselves reported having a disability, even while caring for a person who required assistance with learning, memory, and confusion. Data from one study showed that 36% of caregivers who were aged 65 years or older were considered to be vulnerable, with their health status ranging from fair to poor, and had a serious health condition (29).

In our study, caregivers of people with cognitive impairment reported lower levels of caregiving intensity than did caregivers in the NAC/AARP study (10). However, the construction of the intensity scales differed because we did not ask caregivers the complete list of ADL and IADL. In our study, 62.0% of caregivers of people with cognitive impairment reported they assisted with at least 1 of the categories of ADL-like activities (self-care or moving around), the same percentage of caregivers of people with Alzheimer's disease, dementia, or other mental confusion found in the NAC/AARP study (10). Duration of care was not included in the caregiver intensity variable, but long-term caregiving may contribute to caregiver stress or burden, items not measured in our study. In a study of caregivers of people with Alzheimer's disease, duration of caregiving was not related to caregiver health, when adjusting for behavioral changes in the person receiving care (30). The caregiving intensity measure implies an indirect level of burden or negative impact. A measurement of the positive aspects of caregiving was not captured in our study but may help in future population-based surveillance. One study found that 81% of family caregivers for people with Alzheimer's disease or some other form of dementia reported gains as well as strains associated with their caregiving experience; the remaining 19% reported only burden (31). Previous studies have found mixed results in mental health outcomes for caregivers of people with dementia compared with other caregivers (4,17,19). The results of our study do not indicate any significant differences in frequent mental distress, social support, or life satisfaction between caregivers of people with and without cognitive impairment, which may mean that all caregivers are at equal risk for poor mental health outcomes. Future research is needed to investigate the mental health, including stress and depression, of caregivers.

Our study had several limitations. First, cognitive functioning of the care recipient was not formally assessed. Therefore, care recipients classified as being cognitively impaired may not have had clinical symptoms. Second, there was no indication of the care recipient's severity of cognitive impairment. Previous studies have shown that proxies do not always accurately report disability attributes, such as severity or limitations (32), so proxy assessments of severity of cognitive impairment need validation before inclusion. Third, our data were based on BRFSS respondents in North Carolina, and characteristics of the US population may be different. Future studies should evaluate the possible regional variations in the prevalence of cognitive impairment. Finally, our study included only noninstitutionalized adults (aged ≥18 years) who had traditional home telephone landlines. Despite these limitations, the general attributes of the BRFSS, including its population-based sampling technique and the demonstrated reliability and validity of its core measures (33), allowed comparison of informal caregivers of people with and without cognitive impairment in terms of demographic variables and characteristics of care. Future studies should establish the psychometric properties of the caregiver items, including the abbreviated version of the intensity scale.

The number of caregivers in the United States, including the number of caregivers of people with cognitive impairment, is expected to grow (13). If these caregivers are to continue to provide the foundation of care for people who need assistance, their health, both physical and mental, must be assured. Caregivers, particularly caregivers of people with cognitive impairment, dedicate substantial time to providing care, as our results show. Caregivers of people with cognitive impairment may provide care for long periods of time because of the slow progression of many types of dementia (17). Therefore, caregiving is of public health importance, and caregiving surveillance is vital in assessing and responding to the needs of the growing number of caregivers (5). Evaluating trends in cognitive impairment and caregiving over time is also important. Quantifying the number and type of caregivers in a community will improve our understanding of the health and quality-of-life consequences of providing care and will aid in policy making and decision making.

## Figures and Tables

**Table 1:** Characteristics of Caregivers of People With and Without Cognitive Impairment (Weighted), North Carolina Behavioral Risk Factor Surveillance System, 2005^a^

**Characteristic**	Caregivers of People With Cognitive Impairment (n = 279)	Caregivers of People Without Cognitive Impairment (n = 389)	*P* Value^b^
**Age, y**			
18-34	19.7 (13.0-28.6)	23.5 (15.4-34.1)	.13^c^
35-44	16.0 (11.3-22.1)	18.5 (13.9-24.2)	
45-54	27.8 (21.6-34.8)	19.2 (14.8-24.5)	
55-64	20.8 (15.7-27.1)	17.8 (13.6-22.8)	
≥65	15.8 (11.7-21.0)	21.0 (16.5-26.4)	
**Race/ethnicity**			
Non-Hispanic white	76.7 (70.1-82.2)	74.1 (64.5-81.8)	.63
Non-Hispanic black	15.4 (11.1-21.0)	21.8 (14.3-31.9)	.18
Other/multi-race, non-Hispanic	4.9 (2.5-9.4)	2.4 (1.1-5.2)	.17
Hispanic	3.0 (1.3-6.7)	1.7 (0.6-4.4)	.35
**Sex, female**	59.9 (51.7-67.6)	60.9 (52.2-68.9)	.89
**Education level**			
<High school diploma	6.6 (4.1-10.6)	13.9 (7.3-24.8)	.18
High school diploma	29.6 (22.9-37.4)	29.0 (23.0-35.8)	
>High school diploma	63.7 (56.0-70.8)	57.2 (49.0-65.0)	
**Annual household income, $**			
<25,000	30.3 (23.6-37.9)	23.3 (17.7-30.1)	.10^c^
25,000-34,999	14.8 (9.4-22.6)	20.9 (15.3-27.9)	
35,000-49,999	12.7 (8.6-18.4)	20.5 (12.8-31.1)	
50,000-74,999	21.1 (15.3-28.3)	13.6 (9.8-18.6)	
≥75,000	21.0 (15.0-28.6)	21.7 (16.4-28.1)	
**Health-related quality of life**			
Healthy days in the past 30, mean (95% CI)	24.3 (23.0-25.5)	23.9 (22.4,25.4)	.64^d^
No. of days physical health not good	2.9 (2.1-3.7)	3.3 (2.4-4.3)	.34^d^
No. of days mental health not good	3.9 (2.7-5.0)	3.7 (2.4-5.0)	.81^d^
General health rated fair or poor	16.6 (11.9-22.5)	16.2 (12.3-21.2)	.93
Rarely or never receive social or emotional support	8.7 (5.5-13.7)	6.7 (3.9-11.5)	.47
Dissatisfied or very dissatisfied with life	3.7 (2.0-6.8)	2.7 (1.5-5.0)	.49
**Disability status**			
Have a disability	24.0 (18.6-30.5)	16.1 (12.4-20.8)	.03
Activities limited by physical, mental, or emotional problems	23.4 (18.0-29.9)	15.1 (11.6-19.6)	.02
Use special equipment	7.4 (4.5-12.0)	4.6 (2.7-7.6)	.18

Abbreviation: CI, confidence interval.

a Data are reported as % (95% CI), except where indicated. Numbers may not total to 100% because of rounding.

b Except where indicated, *P* values are reported for the difference in frequencies between caregivers of people with and without cognitive impairment, as measured by χ^2^ test.

c *P* value reported for the difference in frequencies between caregivers of people with and without cognitive impairment, as measured by logistic regression to assess trend across ordinal variables.

d *P* value reported for the difference in means between caregivers of people with and without cognitive impairment, as measured by *t* test.

**Table 2:** Characteristics of Caregiving Experience for Caregivers of People With and Without Cognitive Impairment (Weighted), North Carolina Behavioral Risk Factor Surveillance System, 2005^a^

Characteristic	Caregivers of People With Cognitive Impairment (n = 279)	Caregivers of People Without Cognitive Impairment (n = 389)	*P* Value^b^
**Age of person receiving care, y**			
60-69	10.5 (7.1-15.4)	26.9 (21.3-33.4)	.001^c^
70-79	33.3 (26.3-41.2)	27.9 (22.0-34.7)	
80-89	46.5 (39.0-54.1)	35.6 (27.9-44.1)	
≥90	9.6 (5.5-16.4)	9.6 (6.2-14.5)	
**Sex of person receiving care, female**	74.0 (67.5-79.7)	70.5 (63.4-76.4)	.41
**Relationship of caregiver to person receiving care**			
Spouse/partner	6.3 (3.9-9.9)	10.0 (7.0-14.0)	.12
Other family member	77.4 (70.9-82.8)	69.2 (60.4-76.8)	.07
Nonfamily member	10.9 (7.5-15.7)	18.9 (11.8-28.8)	.07
Paid caregiver	2.7 (1.1-6.4)	0.1 (0.0-0.7)	<.001
**Major health problem of person receiving care**			
Cancer	7.1 (4.5-11.3)	15.7 (11.7-20.7)	.002
Dementia	28.9 (22.0-37.0)	0.6 (0.1-3.0)	<.001
Diabetes	5.9 (3.1-10.7)	10.5 (6.2-17.3)	.14
Heart disease	10.5 (7.0-15.6)	17.8 (13.5-23.1)	.03
Stroke	11.2 (7.3-16.8)	11.3 (7.2-17.3)	.97
**Areas in which person receiving care needs most help**			
Learning, remembering, confusion	37.4 (30.5-44.8)	7.8 (4.2-13.9)	<.001
Seeing or hearing	6.5 (3.9-10.6)	9.4 (6.0-14.5)	.27
Taking care of himself/herself	42.3 (35.1-49.8)	39.2 (31.5-47.6)	.59
Communicating with others	10.3 (6.7-15.6)	7.8 (5.3-11.3)	.32
Moving around	30.5 (23.5-38.6)	51.9 (44.1-59.6)	<.001
Getting along with people	7.1 (4.1-12.1)	4.4 (2.4-7.9)	.24
Feeling anxious or depressed	15.2 (10.8-20.9)	14.9 (11.0-20.0)	.95
**Average hours of care per week, mean (95% CI)**	20.2 (15.2-25.2)	16.6 (12.8-20.4)	.07^d^
**Length of care in months, mean (95% CI)**	45.6 (36.1-55.0)	35.5 (29.6-41.4)	.001^d^
**Caregiving intensity^e^**			
Level 1	31.6 (24.8-39.2)	21.4 (15.7-28.5)	.25^c^
Level 2	34.9 (27.1-43.5)	38.0 (29.6-47.2)	
Level 3	14.6 (10.2-20.5)	20.8 (15.3-27.6)	
Level 4	17.0 (12.4-22.9)	17.3 (12.9-22.7)	
Level 5	2.0 (0.7-5.8)	2.5 (1.3-4.9)	

Abbreviation: CI, confidence interval.

a Data are reported as % (95% CI), except as noted. Numbers may not add to 100% because of rounding.

b Except where indicated, all *P* values are reported for the difference in frequencies between caregivers of people with and without cognitive impairment, as measured by χ^2^ test.

c *P* value reported for the difference in frequencies between caregivers of people with and without cognitive impairment, as measured by logistic regression to assess trend across ordinal variables.

d *P* value reported for the difference in means between caregivers of people with and without cognitive impairment, as measured by *t* test.

e See Methods section for a detailed description of this variable.
